# Bilateral Inguinal Hernia Repair: Laparoscopic Totally Extraperitoneal Versus Open Lichtenstein

**DOI:** 10.15388/Amed.2025.32.2.6

**Published:** 2025-12-30

**Authors:** Mykhaylo O. Yosypenko, Oleg V. Shulyarenko, Hryhorii O. Havrylov

**Affiliations:** 1 Shupyk National Healthcare University of Ukraine, Kyiv, Ukraine; 2 Bogomolets National Medical University, Kyiv, Ukraine; 3 Clinic “Medikom”, Kyiv, Ukraine

**Keywords:** inguinal hernia, laparoscopic totally extraperitoneal repair, Lichtenstein approach, mesh, kirkšnies išvarža, laparoskopinė visiškai ekstraperitoninė išvaržos operacija, Lichtenstein metodas, tinklelis

## Abstract

**Aim:**

To compare the outcome of laparoscopic totally extraperitoneal repair versus the open Lichtenstein technique in the treatment of primary bilateral inguinal hernias.

**Materials and methods:**

The study design was comprised of a matched and randomized research: a total of 93 patients were enrolled in the study and operated in clinic “*Medikom*” from 2015 to 2022. The patients were prospectively randomized and divided into two groups: Group 1 (n=45) underwent TEP repair, whereas Group 2 (n=48) received Lichtenstein repair.

**Result:**

No statistically significant differences were observed between the groups concerning the mean age, sex, body mass index, patient distribution by hernia type, European Hernia Society hernia type, and ASA score (*p*>0.05).

The operating time in Group 1 was on 10.7% more than in Group 2 (*p*<0.05). At 6 hours post-surgery, the pain score in Group 2 was 1.19-fold significantly higher than in Group 1 (*p*<0.05). This significant difference persisted at 24 hours post-surgery, with Group 2 exhibiting a pain score 1.27 times greater than Group 1 (*p*<0.05). The time to resumption of normal activities was 1.5 times longer in Group 2 compared to Group 1, which is a difference that reached statistical significance (*p*<0.05). No statistically significant difference was observed regarding the incidence of early complications between the two groups (*p*>0.05 (χ2-test)). Following a 24-month follow-up period, a total of 42 (93.3%) patients from Group 1 and 45 (93.75%) patients from Group 2 were evaluated. Importantly, neither recurrence nor other complications were observed in either group.

**Conclusions:**

The findings of this trial indicate that laparoscopic total extraperitoneal (TEP) hernia repair offers substantial benefits for patients undergoing bilateral inguinal hernioplasty. The duration until resumption of normal activities was 1.5 times significantly longer for patients in the open hernia repair Group 2 compared to those in the laparoscopic hernia repair Group 1.

## Abstract (it)

**Tikslas:**

Palyginti laparoskopinės visiškai ekstraperitoninės operacijos ir atviros Lichtenstein technikos rezultatus gydant pirmines abipuses kirkšnies išvaržas.

**Medžiagos ir metodai:**

Tyrimas apėmė suderintą ir atsitiktinės atrankos tyrimą: iš viso į tyrimą buvo įtraukti 93 pacientai, kuriems nuo 2015 iki 2022 metų buvo atlikta operacija klinikoje „Medikom“. Pacientai atsitiktinės atrankos būdu suskirstyti į dvi grupes: 1 grupės (n = 45) pacientams buvo atlikta laparoskopinė visiškai ekstraperitoninė operacija, o 2 grupės (n = 48) pacientams taikytas išvaržos operacijos Lichtenstein metodas.

**Rezultatai:**

Nebuvo nustatyta šių grupių pacientų statistiškai reikšmingų skirtumų dėl vidutinio amžiaus, lyties, kūno masės indekso, pacientų pasiskirstymo pagal išvaržos tipą, Europos išvaržų draugijos išvaržos tipą ir ASA balą (p > 0,05).

Operacijos trukmė 1 grupės pacientų buvo 10,7 % ilgesnė nei 2 grupės pacientų (p < 0,05). Praėjus 6 valandoms po operacijos, skausmo balas 2 grupės pacientų buvo 1,19 karto didesnis nei 1 grupės (p < 0,05). Šis reikšmingas skirtumas išliko praėjus 24 valandoms po operacijos, 2 grupės pacientų skausmo balas buvo 1,27 karto didesnis nei 1 grupės (p < 0,05). Laikas iki normalaus gyvenimo atsinaujinimo 2 grupės pacientų buvo 1,5 karto ilgesnis nei 1 grupės pacientų, šis skirtumas buvo statistiškai reikšmingas (p < 0,05). Nėra statistiškai reikšmingo skirtumo tarp dviejų grupių dėl ankstyvo komplikacijų dažnio (p > 0,05(χ2 testas)). Po 24 mėnesių stebėjimo laikotarpio buvo įvertinti 42 (93,3 %) 1 grupės pacientai ir 45 (93,75 %) pacientai iš 2 grupės. Svarbu pažymėti, kad nė vienoje grupėje nebuvo nustatyta recidyvų ar kitų komplikacijų.

**Išvados:**

Šio tyrimo rezultatai rodo, kad laparoskopinė visiškai ekstraperitoninė išvaržos operacija suteikia didelę naudą pacientams, kuriems atliekama abipusės kirkšnies išvaržos operacija. Laikas iki įprasto gyvenimo atsinaujinimo buvo 1,5 karto ilgesnis pacientų, kuriems buvo atlikta atvira išvaržos operacija (2 grupė), palyginti su pacientais, kuriems atlikta laparoskopinė išvaržos operacija (1 grupė).

## Introduction

Inguinal hernia repair stands as one of the most frequently performed surgical procedures globally [1, 2], with over 20 million operations conducted worldwide annually [3].

The epidemiology of hernia development indicates a shifting risk profile, with an increasing incidence observed in younger age groups in addition to older populations, and also a notable sex disparity has been demonstrated [4, 5].

Dreifuss NH [6] reported an incidence of bilateral inguinal hernia reaching 30%.

Inguinal hernia remains a significant surgical challenge due to its high prevalence and notable socioeconomic repercussions, particularly within the economically active population. Furthermore, bilateral cases are associated with extended operative times and an increased economic burden compared to unilateral repairs [6].

Numerous surgical techniques are employed globally for inguinal hernia repair. These methods have undergone continuous evolution, progressing from traditional approaches like Bassini repair to tension-free techniques such as the Lichtenstein procedure, and subsequently to advanced laparo-endoscopic repair techniques. The Lichtenstein procedure, in particular, retains considerable popularity attributed to its simplicity of execution, tension-free principle, and consistently favorable long-term results [7, 8]. According to the international HerniaSurge guidelines for groin hernia management, laparo-endoscopic techniques have less chronic pain and faster recovery than the Lichtenstein repair [9].

## Aim

To compare the outcome of laparoscopic totally extraperitoneal repair versus open Lichtenstein technique in the treatment of primary bilateral inguinal hernias.

## Materials and methods

Study design comprised of a matched and randomized research: a total of 93 patients were enrolled in the study and operated in clinic “Medikom” from 2015 to 2022. The patients were prospectively randomized and divided into two groups: Group 1 (n=45) underwent TEP repair, whereas Group 2 (n=48) received Lichtenstein repair.

Inclusion criteria for this study comprised patients presenting with uncomplicated primary bilateral inguinal hernias and the *American Society of Anesthesiologists* (ASA) physical status classification of I to III. Exclusion criteria encompassed patients with a history of previous preperitoneal surgery (e.g., for hernia, prostate, vascular, or kidney transplant procedures); individuals with strangulated hernias, ascites, or giant scrotal hernias; those presenting with hemostatic disorders; patients exhibiting hemodynamic instability or hypercapnia exceeding 50 Torr; those with a prior laparotomy involving an infra-umbilical extended incision; individuals with severe cardiovascular or respiratory compromise; and septic patients.

Diagnosis of inguinal hernia was established based on a comprehensive medical history, thorough clinical examination, and confirmatory ultrasound imaging. Following the diagnosis, patients underwent pre-anesthesia evaluation and routine diagnostic investigations to ascertain their suitability for anesthesia and surgical intervention.

The participants were divided into two groups: Group 1 (n=45) underwent *Totally Extraperitoneal* (TEP) inguinal hernia repair using our patented method (Patent of Ukraine No. 147109), which incorporated electric bipolar welding hemostasis and a self-gripping lightweight mesh with polypropylene fibers and polylactic acid microhooks. Meanwhile, Group 2 (n=48) received Lichtenstein repair [10], employing electric bipolar welding hemostasis and a standard lightweight mesh. To ensure comparability, the participants in both the open (Lichtenstein) and laparoscopic (TEP) surgical modality groups were carefully matched for potential confounding factors.

We used 10-balls pain *Visual Analog Scale* (VAS). All patients were given same antibiotics and pain relief, anticoagulation medications, which was also implemented for correction of cardiovascular and respiratory disorders so that to maintain uniformity across the groups. The patients were advised to take non-opioid (Ketorolac), and, in case of pain more then 4 (VAS) balls, opioid (Omnopon) analgesics.

Postoperative follow-up was conducted at 6 and 24 hours while the patients were in the inpatient department, on postoperative day 7 at the outpatient department (OPD), and subsequently via telephone at 3 months, 6 months, and 2 years post-surgery. The patients were queried regarding the presence of pain and recurrent swelling. Individuals reporting these symptoms were subsequently recalled to the OPD for a comprehensive physical examination.

Table 1 summarizes the baseline patient demographics and distribution across both study groups.

**Table 1:** Distribution of baseline characteristics of the study participants among study groups

		Group 1 (TEP repair) (n=45)	Group 2 (Lichtenstein repair) (n=48)	*p*-value
Mean age, years		49.89±0.97	51.61±0.99	0.217
Gender	male, n	32	34	0.977
	female, n	13	14	
Type of hernia	direct, n	10	11	0.995
	undirect, n	32	34	
	both, n	3	3	
European Hernia Society hernia type	M1P, n	2	3	0.508
	M2P, n	5	6	
	M3P, n	3	2	
	L1P, n	5	5	
	L2P, n	22	23	
	L3P, n	5	6	
	M2P+L2P, n	2	2	
	M2P+L3P, n	1	1	
ASA, class	I	12	13	0.619
	II	30	29	
	III	3	6	
Body mass index, kg/m^2^		23.92±0.51	25±0.37	0.089

The operative time, postoperative pain severity at 6 hours, 24 hours, and 7 days (assessed by using a 10-point Visual Analog Scale (VAS) score), the incidence of postoperative complications over a 24-month follow-up period, and the mean time to resumption of normal daily activities served as the primary outcome measures for this study.

All statistical analyses were performed by using a dedicated software package. Student’s t-test was employed to compare continuous variables, including age, body mass index, operative time, postoperative pain severity, and time to return to normal activities. Quantitative data are expressed as mean ± standard deviation (M±m). A value of *p*<0.05 was considered statistically significant. Categorical data, such as the patient distribution by sex, hernia type, European Hernia Society hernia type, ASA class, and incidence of early postoperative complications, were analyzed by using the Chi-square (χ2) test. For these analyses, *p*<0.05 was set as the threshold for statistical significance.

## Results

Table 1 demonstrates no statistically significant differences between the two groups regarding the mean age, sex, body mass index (BMI), patient distribution by hernia type, European Hernia Society hernia type, or ASA physical status classification (p>0.05). This confirms the comparability of the two cohorts

The operative time was defined as the interval from the initial skin incision to the final skin closure.

As shown in Table 2, the operative time in Group 1 was 10.7% longer than in Group 2, which is a difference that was statistically significant (*p*<0.05). This finding indicates that the totally extraperitoneal (TEP) technique, on average, requires more operative time compared to the open Lichtenstein technique.

**Table 2:** Outcome parameters assessed and significance of difference between the two groups

		Group 1 (TEP repair) (n=45)	Group 2 (Lichtenstein repair) (n=48)	*p*-value
operating time, min		109.57±1.39	98.95±0.9	<0.0001
Pain scorepost op (VAS), balls	6 hours	5.51±0.08	6.53±0.08	<0.001
	24 hours	2.57±0.08	3.26±0.07	<0.001
	7^th^ day	2.11±0.05	2.58±0.08	<0.001
Port-site seroma, case		1	0	0.36
Postoperative wound hematoma, case		0	1	
Urinary retention, case		1	1	
Time until return to normal activities, days		12.8±0.13	19.32±0.11	<0.001

The pain score 6 hours after surgery in Group 2 was in 1.19 times significantly higher comparing with Group 1 (*p*<0.05). The pain score 24 hours after surgery in Group 2 was 1.27 times significantly higher if comparing with Group 1 (*p*<0.05). The pain score 7 days after surgery in Group 2 was in 1.22 times significantly higher comparing with Group 1 (*p*<0.05).

In Group 1, port-site seroma developed in 1 case (2.22%); it was successfully punctured under sonography control.

In Group 2, a wound-site hematoma developed in 1 case (2.08%), it was successfully coagulated by using a bipolar device.

We report no case of the operation which would have been converted to a different type of repair.

The duration until resumption of normal activities was 1.5 times significantly longer for patients in Group 2 compared to Group 1 (*p*<0.05).

There was no case of major complication in either group.

No significant difference was reported concerning the incidence of early complications between the two groups (*p*>0.05 (χ2-test)).

Following a 24-month follow-up period, a total of 42 (93.3%) patients from Group 1 and 45 (93.75%) patients from Group 2 were evaluated. Importantly, neither recurrence nor other complications were observed in either cohort.

## Discussion

The myopectineal orifice (MPO) is a crucial anatomical landmark [11], bounded superiorly by the conjoined tendon, inferiorly by Cooper’s ligament, medially by the rectus abdominis muscle and its sheath, and laterally by the iliopsoas muscle. Within its confines lie Hesselbach’s triangle, the femoral canal, and the deep inguinal ring.

While both TEP and Lichtenstein hernioplasties are tension-free procedures, laparoscopic hernia repair has demonstrated short-term superiority over the open Lichtenstein technique [12, 13]. This advantage is primarily attributed to its minimal incision length, leading to shorter convalescence and sick leave durations, in addition to reduced postoperative pain. However, laparoscopic mesh repair has faced criticism primarily due to its inherent technical complexity, compounded by associated complications during the early phase of the learning curve [14]; consequently, reported outcomes remain conflicting. Furthermore, the laparoscopic approach necessitates general anesthesia, is inherently more complex and challenging to master than the open procedure, and incurs higher intra-hospital costs [15, 16].

In the current study, no recurrence was observed in either group after a 2-year follow-up period. The recurrence rates for both Lichtenstein and TEP procedures reported herein are consistent with findings from numerous other series [17, 18], which similarly indicate low recurrence rates during the postoperative follow-up.

Early recurrences (within the first year) reported in previous studies [7, 19] have been attributed to factors such as the inadequate mesh size, mesh displacement, or an incorrect surgical technique.

This study demonstrates that laparoscopic inguinal hernia repair (LIHR) via the TEP technique, by utilizing a semi-absorbable self-fixating mesh, is a rapid, effective, and reliable method. It effectively combines the inherent advantages of the laparoscopic approach with the straightforward and practical implantation of a self-fixating mesh. Our findings indicate that this method significantly reduces both complication and recurrence rates [20, 21, 22].

In contrast to open Lichtenstein repair, where the mesh is placed anteriorly, laparoscopic repair involves preperitoneal mesh placement. This allows for comprehensive coverage of the entire myopectineal orifice from the inside, thereby ensuring a tension-free repair without the need for suturing local tissues; this coverage extends to both the femoral and inguinal openings. The strategic placement of the prosthetic mesh in the preperitoneal space not only restores the integrity of the transversalis fascia but also constitutes the fundamental principle of current laparoscopic herniorrhaphy techniques [23].

We are in agreement with Koprivica [24] that the TEP procedure is advantageous as its mesh reinforcement avoids entering into the peritoneal cavity, especially in case of bilateral inguinal hernia repair.

For patients presenting with bilateral or recurrent inguinal hernias, laparoendoscopic repair offers significant advantages over open techniques, particularly concerning postoperative pain, recurrence risk, and recovery time. In cases of bilateral hernias, both sides can be addressed through the same access and port placement, and laparoscopy facilitates complete intra-abdominal visualization [25, 26, 27, 28].

## Limitations of the study

Despite the compelling results, it is imperative to acknowledge the limitations of this work. Firstly, this study was conducted at a single center, which may limit the generalizability of our findings to other populations or healthcare settings. Secondly, while our sample size of 93 patients provided sufficient power for the observed differences, future studies with larger cohorts would further strengthen the evidence. Additionally, the 24-month follow-up period is a reasonable duration for assessing recurrence and early complications, but a longer-term follow-up would provide more comprehensive data on chronic pain and very late recurrences. Future research should aim for multi-center, prospective, randomized controlled trials with extended follow-up periods to validate these findings and explore additional patient-reported outcomes.

## Conclusions

1. The current trial unequivocally underscores the substantial advantages of laparoscopic total extraperitoneal (TEP) hernia repair for individuals requiring bilateral inguinal hernioplasty
2. Patients undergoing open hernia repair (Group 2) experienced a statistically significant 1.5-fold longer duration until resumption of normal activities compared to those in the laparoscopic hernia repair group (Group 1) (*p*<0.05).

## References

[1] Takayama Y, Kaneoka Y, Maeda A, Takahashi T, Uji M (2020). Laparoscopic transabdominal preperitoneal repair versus open mesh plug repair for bilateral primary inguinal hernia. *Ann Gastroenterol Surg*.

[2] Shukur NM (2024). Study on evaluation of effectiveness of pre-peritoneal mesh repair for bilateral and recurrent inguinal hernia. *Int J Med Pub Health*.

[3] Hidalgo NJ, Guillaumes S, Bachero I, Holguín V, Momblán D (2023). Trends and predictors of laparoscopic bilateral inguinal hernia repair in Spain: a population-based study. *Surg Endosc*.

[4] Hoffmann H, Mechera R, Nowakowski D, Adolf D, Kirchhoff P, Riediger H, Köckerling F (2023). Gender differences in epigastric hernia repair: A propensity score matching analysis of 15,925 patients from the herniamed registry. *Hernia*.

[5] Perez AJ, Campbell S (2022). Inguinal hernia repair in older persons. *J Am Med Dir Assoc*.

[6] Dreifuss NH, Pena ME, Schlottmann F, Sadava EE (2021). Long-term outcomes after bilateral transabdominal preperitoneal (TAPP) repair for asymptomatic contralateral inguinal hernia. *Surg Endosc*.

[7] Feleshtinsky YY, Kohanevich AV (2019). Estimation of options of the mesh implant fixation in transabdominal preperitoneal alloplasty in patients with inguinal hernia. *Med perspekt*.

[8] Messias BA, Nicastro RG, Mocchetti ER (2024). Lichtenstein technique for inguinal hernia repair: ten recommendations to optimize surgical outcomes. *Hernia*.

[9] Stabilini C, van Veenendaal N, Aasvang E (2023). Update of the international HerniaSurge guidelines for groin hernia management. *BJS Open*.

[10] Lichtenstein IL, Shulman AG, Amid PK (1991). Twenty questions about hernioplasty. *Am Surg*.

[11] Bisciotti GN, Bisciotti A, Auci A, Bisciotti A, Volpi P (2024). Anatomical Features in Inguinal-Pubic-Adductor Area That May Contribute to Gender Difference in Susceptibility to Groin Pain Syndrome. *J Pers Med*.

[12] Xie J, Koo DC, Lee MJ, Sugiyama G (2024). The evolution of minimally invasive inguinal hernia repairs. *Ann Laparosc Endosc Surg*.

[13] Lillo-Albert G, Villa EB, Boscà-Robled A (2024). Chronic inguinal pain post-hernioplasty. Laparo-endoscopic surgery vs lichtenstein repair: systematic review and meta-analysis. *Hernia*.

[14] Olanrewaju OA, Saleem A, Owusu FA, Pavani P, Ram R, Varrassi G (2023). Contemporary Approaches to Hernia Repair: A Narrative Review in General Surgery. *Cureus*.

[15] Ko H, Lee SM, Chang HK, Min SY, Cho K, Park MS (2023). Laparoscopic total extra-peritoneal (TEP) inguinal hernia repair under local anesthesia by topical lidocaine injection. *Hernia*.

[16] Sivakumar J, Chen Q, Hii MW (2023). Learning curve of laparoscopic inguinal hernia repair: systematic review, meta-analysis, and meta-regression. *Surg Endosc*.

[17] Lillo-Albert G, Villa EB, Boscà-Robledo A (2024). Chronic inguinal pain post-hernioplasty. Laparo-endoscopic surgery vs lichtenstein repair: systematic review and meta-analysis. *Hernia*.

[18] Haladu N, Alabi A, Brazzelli M (2022). Open versus laparoscopic repair of inguinal hernia: an overview of systematic reviews of randomised controlled trials. *Surg Endosc*.

[19] Wantz GE (1998). Giant prosthetic reinforcement of the visceral sac. The Stoppa groin hernia repair. *Surg Clin North Am*.

[20] Okamoto N, Mineta S, Mishima K (2023). Comparison of short-term outcomes of robotic and laparoscopic transabdominal peritoneal repair for unilateral inguinal hernia: a propensity-score matched analysis. *Hernia*.

[21] Nanayakkara K, Viswanath NG, Wilson M (2023). An international survey of 1014 hernia surgeons: outcome of GLACIER (global practice of inguinal hernia repair) study. *Hernia*.

[22] Sharma K, Koul A, Puri G, Rathore YS, Chrungoo RK (2024). Comparison of modified tumescent and conventional laparoscopic transabdominal pre-peritoneal repair in the patients of inguinal hernia: A randomised control trial. *J Minim Access Surg*.

[23] Nagata S, Orita H, Korenaga D (2023). Nonfixation of mesh in laparoscopic totally extraperitoneal inguinal hernia repair: A propensity score matched analysis. *Asian J Surg*.

[24] Koprivica R, Perišić S, Čopi J, Šadl J (2024). Totally extraperitoneal versus transabdominal preperitoneal laparoscopic techniques for hernia inguinal repair using glue for mesh and peritoneal closure. *Medical Research Archives*.

[25] Pohnán R, Rozwadowski F, Klein L, Ryska M (2013). Advantages and disadvantages of transabdominal preperitoneal approach and total extraperitoneal approach versus open repair of inguinal hernia. *Mil Med Sci Lett*.

[26] Ferri V, Vicente E, Quijano Y (2023). OC-067 Cost-effectiveness and clinical outcomes analysis of laparoscopic total extraperitoneal (tep) and laparoscopic trans-abdominal preperitoneal (tapp) inguinal hernia repair. *BJS*.

[27] Ungureanu CO, Ginghina O, Stanculea F (2023). Surgical Approach to Bilateral Inguinal Hernia. A Case-Control Study. *Chirurgia (Bucur)*.

[28] Chamzin A, Theodoropoulos C, Galyfos G, Triantafyllou A, Michalopoulos NV, Toutouzas KG, Theodorou D (2024). Laparoscopic tapp repair of spigelian and bilateral inguinal hernia. *BJS*.

[29] Sakoğlu N, Gök MA, Civil O (2024). Effectiveness and Results of the Laparoscopic Total Extraperitoneal Repair (Tep) Method Applied in Inguinal Hernia Surgery: 10 Years of Experience. *Dicle Tıp Dergisi*.

[30] Syeda Saima Qamar Naqvi, Sidra Abbas, Hammad Hanif, Rizwan Ahmed Khan, Syed Mohammad Abdullah Bukhari, Syed Adnan Ahmed (2023). Open Mesh Hernioplasty versus Laparoscopic Total Extraperitoneal Mesh Repair in the Treatment of Inguinal Hernia. *Pak J Med Health Sci*.

